# The Kefallinia Score: Development and Apparent Performance Assessment of a Flow Cytometry-Based Intraoperative Classification System for Intracranial Tumors

**DOI:** 10.3390/cancers18213547

**Published:** 2026-11-04

**Authors:** Georgios S. Markopoulos, Alexandros Liaropoulos, Evrysthenis Vartholomatos, Konstantinos Liaropoulos, Spyridon Voulgaris, George Alexiou, George Vartholomatos

**Affiliations:** 1 Haematology Laboratory, Unit of Molecular Biology and Translational Flow Cytometry, 45110 Ioannina, Greece; gmarkop@uoi.gr; 2 Neurosurgical Institute, University of Ioannina, 45110 Ioannina, Greece; eyrys.varth@gmail.com (E.V.); svoulgar@uoi.gr (S.V.); galexiou@uoi.gr (G.A.); 3 Department of Physiology, Faculty of Medicine, School of Health Sciences, University of Ioannina, 45110 Ioannina, Greece; 4 Neurosurgical Department, Athens Euroclinic, 11521 Athens, Greece; aliaropoulosabc@gmail.com (A.L.); kliarop@gmail.com (K.L.); 5 Department of Neurosurgery, University Hospital of Ioannina, 45110 Ioannina, Greece

**Keywords:** intraoperative flow cytometry, brain tumors, glioma, meningioma, lymphoma, metastasis, tumor index, DNA index, neurosurgery

## Abstract

Background: Intraoperative flow cytometry (iFC) provides rapid quantitative information on DNA content, cell-cycle distribution, tumor index, ploidy, and selected immunophenotypic features during tumor surgery. Existing iFC studies in neurosurgery have mainly focused on glioma grading, meningioma grade assessment, margin evaluation, or specific binary diagnostic questions. In this study, we evaluated the Kefallinia Score as an iFC-based classification system for broad diagnostic orientation in neurosurgery. Methods: We retrospectively analyzed a de-identified, complete-panel dataset from 90 patients with intracranial tumors. The score combined DNA-index abnormality, tumor index, an operational low-DNA-content fraction, CD20/CD45/CD19 positivity, cytokeratin positivity, and glial fibrillary acidic protein (GFAP) expression. Score intervals assigned cases to low-grade/meningioma-predominant (0–4), glioma-spectrum (5–14), lymphoma-oriented (15–29), or metastasis-oriented (≥30) categories. The scoring rules were defined before specimen collection and evaluated retrospectively against final routine pathological diagnoses. Results: The cohort included 41 low-grade/meningioma-predominant lesions, 36 glioma-spectrum tumors, two lymphomas, and 11 metastases. Apparent accuracy was 90.0% (81/90; 95% CI, 81.9–95.3%) and balanced accuracy was 92.85% (95% stratified bootstrap CI, 87.1–97.6%). Sensitivity/specificity were 80.5%/98.0%, 100.0%/87.0%, 100.0%/98.9%, and 90.9%/100.0%, respectively. Conclusions: These findings support further evaluation of the classification framework to assess possible future clinical use.

## Simple Summary

Ιntraoperative flow cytometry rapidly measures cellular features that may help characterize tumors during surgery. We evaluated the Kefallinia Score, a predefined scoring system that combines DNA measurements and selected cellular markers to support diagnostic classification for intracranial tumors. The score assigns cases to four categories: low-grade/meningioma-predominant, glioma-spectrum, lymphoma-oriented, and metastasis-oriented. In a retrospective study of 90 patients, the assigned category agreed with the category based on the final pathological diagnosis in 81 cases (90.0%). These findings suggest that combining several flow cytometry measurements may support intraoperative tumor classification. However, the results reflect performance within the studied cohort, and some tumor groups were small, particularly lymphomas. Further evaluation in larger, independent cohorts is needed to assess possible future clinical use.

## 1. Introduction

Maximal safe resection remains a central objective in neurosurgical oncology. In gliomas, the extent of resection is closely related to clinical outcome, but surgical aggressiveness must be balanced against the risk of neurological comorbidities. In meningiomas, recognition of higher-grade biological behavior during surgery may influence the extent of resection, clinical management and postoperative surveillance. Similarly, intraoperative distinction among glioma, lymphoma, metastasis, and low-grade or meningioma-predominant lesions may affect whether the surgeon proceeds with extensive cytoreduction, biopsy, margin sampling, or tissue preservation [1].

The 2021 World Health Organization classification of central nervous system tumors emphasizes integrated histopathological and molecular diagnosis, especially in diffuse gliomas, where IDH status and 1p/19q codeletion define major diagnostic entities [2]. Conventional intraoperative pathology remains the gold standard for immediate tissue diagnosis. However, final integrated diagnosis often depends on tissue processing, immunohistochemistry, and molecular testing that cannot always be completed during the operative window.

Intraoperative flow cytometry has emerged as a rapid adjunct capable of quantifying DNA content, cell-cycle distribution, proliferative fraction, ploidy, and selected immunophenotypic markers from fresh tissue [3]. In CNS tumors, the field has been shaped by two complementary traditions: the Japanese experience based on malignancy index and ultrarapid DNA-content analysis, and the Ioannina experience based on rapid cell-cycle and ploidy assessment using the Ioannina Protocol [4].

The Tokyo, Japan group demonstrated that iFC could rapidly detect glioma tissue and provide information on histopathological grade. Shioyama and colleagues showed strong diagnostic performance for tumor identification using a malignancy index, with an ROC-AUC of 0.941 and a cutoff of 6.8% of analyzed cells having greater-than-normal DNA content for identifying tumors in surgical biopsy specimens [5]. Subsequent Japanese studies extended iFC to low-grade glioma prognosis, glioblastoma prognosis, meningioma malignancy assessment and differentiation of primary central nervous system lymphomas from glioblastomas [6,7,8,9]. Koriyama and colleagues specifically evaluated iFC for differentiating primary central nervous system lymphomas from glioblastomas [8].

In parallel, the Ioannina, Greece group developed a fast cell-cycle analysis workflow that provides information on G0/G1, S-phase, G2/M fraction, tumor index, and DNA ploidy within minutes. Early work showed that rapid cell-cycle analysis could differentiate low- from high-grade tumors and contribute to characterization of glioma margins [10]. The approach was also evaluated in pediatric brain tumors, supporting the broader feasibility of rapid flow-cytometric cell-cycle assessment in neurosurgical specimens [4].

More recently, the Ioannina Protocol was applied specifically to glioma grading and margin assessment. In the study by Vartholomatos and colleagues, iFC enabled tissue analysis within 5–6 min and assessed G0/G1 phase, S-phase, mitosis, tumor index, and ploidy status. High-grade gliomas had a significantly higher tumor index than low-grade gliomas, and iFC verified malignant tissue in glioma margin samples using histology as the reference standard [11]. The same conceptual framework was applied to meningiomas, where iFC distinguished grade 1 from grade 2/3 tumors, and ROC analysis identified a tumor-index cutoff of 11.4% for discriminating low- from high-grade meningiomas [12]. More recently, metastatic brain tumors have been discriminated from glioblastomas with 75% accuracy, evaluating only DNA-content characteristics [13].

Together, these studies support iFC as a rapid quantitative adjunct for intraoperative CNS tumor assessment. However, most existing work addresses specific binary or near-binary questions, such as low- versus high-grade glioma, low- versus high-grade meningioma, tumor versus margin, or lymphoma versus glioblastoma. In routine neurosurgical practice, the intraoperative question is often broader: whether the tissue profile is more compatible with a low-grade/meningioma-predominant lesion, a glioma/glioblastoma-spectrum tumor, a lymphoma-oriented profile, or metastatic epithelial malignancy. The present study introduces the Kefallinia Score, an intraoperative flow cytometry-based classification system for neurosurgery. The score integrates DNA-index abnormality, tumor index, an operational low-DNA-content fraction, an indication of necrosis, lymphoid/hematopoietic marker positivity, cytokeratin positivity, and GFAP expression into a single rule-based framework. We retrospectively evaluated the diagnostic performance of the Kefallinia Score in a pooled dataset derived from prior and ongoing work of the Ioannina intraoperative flow cytometry group.

## 2. Materials and Methods

### 2.1. Study Design

This study was a retrospective assessment of iFC-derived data from intracranial tumor cases evaluated by the Ioannina intraoperative flow cytometry group. The Kefallinia Score, including its component weights and category intervals, was defined before specimen collection began. The present analysis assessed the performance of this predefined rule in the available complete-panel cohort. No independent external validation was performed.

The score was designed as a pragmatic diagnostic-orientation system integrating DNA-content/cell-cycle information with selected immunophenotypic and lineage-associated markers. Its intended application is intraoperative classification; the present analysis did not evaluate and had no impact on surgical decision-making.

The present analysis was based on de-identified data generated within the framework of previously published studies by our group on iFC in gliomas and meningiomas, together with additional intracranial tumor cases evaluated using the same institutional workflow [11,12]. No additional patient intervention, tissue sampling, or experimental procedure was performed specifically for the present analysis. This pooled institutional cohort is not an independent external-validation sample.

### 2.2. Source Dataset

The dataset comprised 90 intracranial tumor specimens from 90 different patients evaluated between 5 January 2021 and 29 December 2023 within institutional iFC study. These were cases with the complete panel required to calculate all six score components. Inclusion was based on panel completeness rather than score result or agreement with pathology. Case IDs 1–90 in Supplementary Table S1 identify the included patients. The finalized analysis table contains the verified component points and reference groups used for all calculations. Original continuous measurements and event-level flow files were not re-extracted from each specimen for this retrospective score analysis. The cohort therefore only represents patients with all available complete panel components and does not establish performance in patients with incomplete or failed assays.

### 2.3. Reference Standard and Diagnostic Harmonization

Reference-group assignment followed the final routine pathological diagnosis recorded for each patient. Diagnoses were grouped into four mutually exclusive categories: low-grade/meningioma-predominant lesions, glioma-spectrum tumors, lymphoma, and metastasis. The investigators checked these assignments against the clinical diagnoses and retained them throughout the analysis, independently of score performance. The first category is descriptive rather than restricted to a single WHO grade; case 39, for example, was an atypical meningioma, WHO grade II. The analysis concerns broad tumor classification and does not replace final histological or integrated molecular diagnosis.

### 2.4. Cell-Cycle, DNA-Content, and Marker Analysis

DNA-content and cell-cycle measurements followed the previously described Ioannina Protocol. The principal cell-cycle variable used in the Kefallinia Score was the tumor index, defined as the cumulative percentage of cells in S phase and G2/M phase. This variable reflects the proliferative fraction of the analyzed tumor sample and has previously been associated with tumor grade and malignant biological behavior in intracranial tumors [11,12]. The workflow is summarized in Supplementary Methods S1.

DNA index was used as a marker of DNA-content abnormality and aneuploidy. It was defined as the ratio of the geometric mean fluorescence intensity of the G0/G1 tumor-cell population to that of normal diploid control cells. In the Kefallinia Score dataset, DNA-index abnormality was entered as a categorical score component with two categories. DNA index < 0.9 or >1.1 received 4 points, whereas 0.9 ≤ DNA index ≤ 1.1 received 0 points.

The operational low-DNA-content fraction retained cellular events with DNA fluorescence below the G0/G1 population. This component may indicate necrosis after exclusion of events interpreted as cellular debris. A fraction > 10% received 2 points and ≤10% received 0 points. The cutoff was empirical, since no independent cell-death assay was performed to distinguish necrosis from apoptosis or processing effects. This component is therefore described as a low-DNA-content fraction rather than confirmed necrosis.

Marker expression was assessed by flow cytometry as described in Supplementary Methods S1. Representative marker histograms are shown in Supplementary Figure S1. Laboratory measurements and marker interpretation preceded disclosure of the final pathological diagnosis to the operators. The verified component-point table was retained as the final dataset. No additional tumor-specific gating or reactive-cell exclusion procedure was inferred from the point table.

### 2.5. Kefallinia Score Construction

The Kefallinia Score was calculated as the sum of six components: DNA-index component + tumor-index component + low-DNA-content component + lymphoid-marker component + cytokeratin component + GFAP component. The operators defined the components, weights and category intervals empirically before specimen collection began, based on the intended classification framework. These choices were not fitted to the results of the present 90 cases. DNA index, TI, low-DNA-content fraction and GFAP contribute at most 4 + 3 + 2 + 5 = 14 points. In the absence of cytokeratin positivity, a positive lymphoid component adds 15 points and places the score within 15–29; cytokeratin positivity adds 30 points and places it at ≥30. These design constraints explain the category hierarchy, but the optimality of the individual weights and measurement thresholds was not established. All rules were applied unchanged throughout the retrospective performance assessment. The GFAP intervals use the continuous boundaries in Table 1 without an additional rounding rule.

Total score was mapped to the four categories using the ranges in Table 1. The intervals classify recorded profiles and should not be interpreted as tumor-grade thresholds.

### 2.6. Apparent Classification Performance and ROC Analysis

The fixed Kefallinia Score categories were compared with the recorded reference diagnostic groups. A four-class confusion matrix was generated. Overall accuracy and balanced accuracy, defined as the unweighted mean of the four category-specific sensitivities, were calculated. For each category, sensitivity, specificity, positive predictive value, and negative predictive value were reported with their numerator/denominator counts and exact binomial 95% confidence intervals (Clopper–Pearson). Predictive values reflect the diagnostic distribution of this selected cohort and are not transportable to other settings without reassessment. The success flag entries were used only as a consistency check against the calculated classifications.

Receiver operating characteristic analysis was performed as an exploratory secondary analysis. Because the score is a rule-based taxonomy with terminal monotonic classes and interval-defined intermediate classes, category-specific evidence scores were used. For low-grade/meningioma-predominant lesions, lower scores were treated as stronger evidence; for metastasis, higher scores were treated as stronger evidence. For gliomas and lymphomas, interval-proximity evidence scores were used for {5,14} and {15,29}, respectively. For an interval {a,b}, evidence was defined as −max(a − S, 0, S − b), where S is the total score; all scores within an interval received equal evidence. AUCs describe apparent discrimination in the same cohort, not internal validation.

The fixed category assignments were the primary analysis. AUC confidence intervals were obtained from 50,000 stratified one-vs-rest bootstrap replicates using the 2.5th and 97.5th percentiles (random seed 20261004 plus category index, 0–3).

Statistical analyses and statistical figure generation were performed in Python 3.12.14. The results were independently cross-checked using IBM SPSS Statistics, version 26.

### 2.7. Ethics

The study was conducted in accordance with the Declaration of Helsinki within the institutional iFC study approved by the Institutional Review Board of the University Hospital of Ioannina (protocol code 73/5-1-2021; approved 5 January 2021), which also provided the framework for previously published studies [11,12]. The 90 cases, including the additional cases and the present secondary analysis, were included within this parent study. Informed consent was obtained under the parent study protocol. This analysis used de-identified records and involved no additional tissue sampling, patient contact, or therapeutic intervention.

## 3. Results

A total of 90 records with complete component points and reference-group labels were included: 41 low-grade/meningioma-predominant lesions, 36 glioma-spectrum tumors, two lymphomas, and 11 metastases. Total scores, predicted categories, and correctness flags were independently calculated and were consistent in all 90 records. TI points were 2 in 36 cases and 3 in 54 cases; no record had zero TI points. The investigators confirmed that none of these 90 specimens had TI < 6%; zero TI points were therefore not observed. This distribution does not establish that TI < 6% excludes malignancy in other samples.

The Kefallinia Score showed distinct distribution patterns across diagnostic groups. Low-grade/meningioma-predominant lesions had a median score of 4 with a range of 2–22. Glioma-spectrum tumors had a median score of 8 with a range of 5–14. Lymphomas had scores of 17 and 22, while metastases had a median score of 36 with a range of 3–39. Score distributions are summarized in Supplementary Table S2.

Using the fixed score intervals, the predicted diagnostic distribution was 34 low-grade/meningioma-predominant lesions, 43 glioma-spectrum tumors, three lymphomas, and 10 metastases. The score correctly classified 81/90 cases, corresponding to an apparent accuracy of 90.0% (95% CI, 81.9–95.3%) and balanced accuracy of 92.85% (95% stratified bootstrap CI, 87.1–97.6%). The balanced-accuracy interval is conditional on the observed groups and fixed rules; in particular, bootstrap resampling cannot compensate for only two lymphoma cases. The confusion matrix and balanced-accuracy interval are provided in Supplementary Table S3, and category-specific performance is summarized in Table 2.

Nine cases were misclassified. Seven low-grade/meningioma-predominant records (59, 85–90) entered the glioma interval; their scores were 12, 8, 5, 5, 5, 7, and 10. Case 39 was an atypical meningioma, WHO grade II (WHO 2016), with borderline CD45 and CD20 positivity around the laboratory cutoff. Its 4 DNA points, 3 TI points and 15 lymphoid-marker points yielded a total of 22 and a false lymphoma classification. Case 83, confirmed in the metastatic reference group, had 3 TI points and zero points for every other component (total 3). The investigators rechecked the source diagnoses and measurements for the misclassified cases and retained the original labels and points. Supplementary Table S4 shows all six components and the available case-specific clarification. The numerical routes to error are reproducible, whereas the biological or technical mechanisms remain to be investigated in larger cohorts.

**Figure 1:**
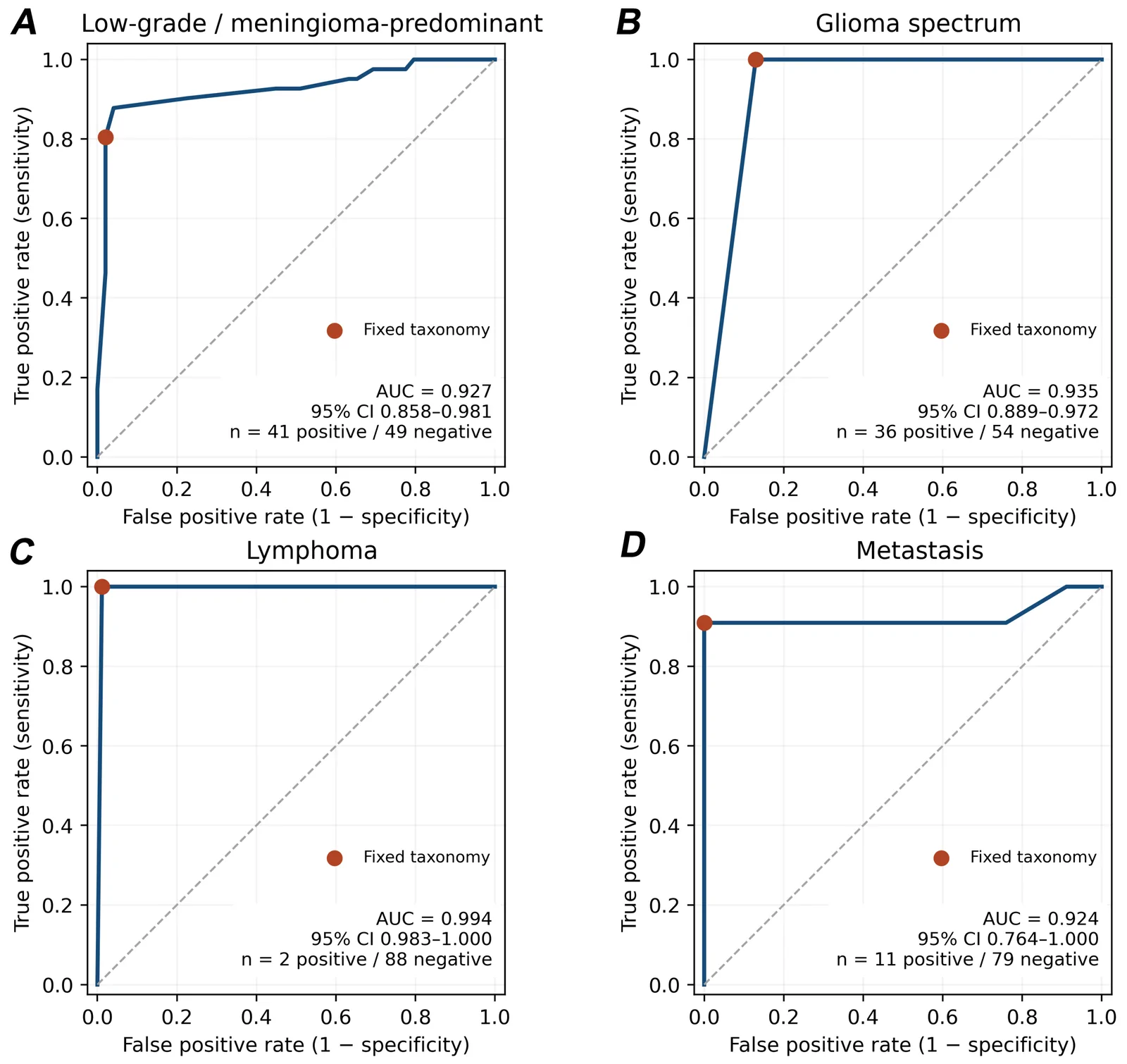
Exploratory one-vs-rest ROC analysis of the Kefallinia Score. (**A**) Low-grade/meningioma-predominant lesions; (**B**) glioma-spectrum tumors; (**C**) lymphoma; and (**D**) metastasis. Evidence was −S for A, S for (**D**), and −max(a − S, 0, S − b) for (**B**,**C**), using {5,14} and {15,29}, respectively. Dots indicate the fixed category operating points (0–4, 5–14, 15–29, ≥30). AUCs and 95% stratified bootstrap intervals are displayed in each panel. For (**B**,**C**), all positive cases lie within their corresponding interval, so AUC equals the mean of sensitivity and specificity at the fixed point and adds no independent validation. Lymphoma estimates are particularly uncertain because only two positive cases were included. The curves and intervals describe apparent performance.

Exploratory one-vs-rest ROC analysis showed high apparent discrimination. The glioma and lymphoma categories are interval-defined rather than monotonic functions of total score, and their ROC results do not independently validate the fixed classifier. The curves and operating points are shown in Figure 1A–D; AUCs and bootstrap intervals are provided in Supplementary Table S5.

The marker-only comparator correctly classified 78/90 cases (86.7%; balanced accuracy 90.5%), compared with 81/90 for the full score. The paired accuracy difference was 3.3 percentage points (95% stratified bootstrap CI, −2.2 to 8.9; exact McNemar *p* = 0.508). Six cases were correct only with the full score and three only with the marker-only rule. DNA/cell-cycle points alone correctly classified 54/90 cases (60.0%). Removing DNA or low-DNA-content points separately left all 90 full-score predictions unchanged. Removing TI points changed six predictions but retained 81/90 correct. These exploratory comparisons do not establish incremental clinical benefit (Supplementary Table S6).

## 4. Discussion

The present study introduces the Kefallinia Score, a rule-based framework combining DNA-content and lineage-associated marker information for broad neurosurgical classification. Apparent accuracy was 90.0% and balanced accuracy 92.85% in the available retrospective cohort. Glioma sensitivity was 36/36 and metastasis specificity 79/79, but these same-cohort estimates should not be interpreted as established clinical performance. The findings support the initial classification performance of the predefined framework, while prospective implementation and clinical utility require independent evaluation. To our knowledge, this is the first flow cytometry-based framework that supports intraoperative classification analysis, which is the main advantage of the current study.

The rationale of the Kefallinia Score is based on previous work showing that iFC captures biologically relevant features of intracranial tumors during the operative window. In our previous glioma study, tumor index was significantly higher in high-grade than low-grade gliomas, a tumor-index cutoff of 17% differentiated low- from high-grade gliomas with high specificity, and iFC identified malignant tissue in glioma margin samples using histology as the reference standard [11]. In the corresponding meningioma study, iFC distinguished grade 1 from grade 2/3 tumors, with higher-grade tumors showing higher S-phase fraction, G2/M fraction, and tumor index, and ROC analysis identified a tumor-index cutoff of 11.4% for discriminating low- from high-grade meningiomas [12]. The present study builds on this logic to ask a broader question: whether iFC can support rapid orientation among major intraoperative neurosurgical diagnostic entities.

The main conceptual advantage of the Kefallinia Score is interpretability. The tumor index reflects proliferative activity, whereas DNA-index abnormality captures gross DNA-content abnormality. These features are biologically related to malignant behavior [14]. Necrosis is relevant to the glioma microenvironment [15,16], although the low-DNA-content fraction used here was not validated as a specific measure of necrosis and may also reflect apoptosis or processing effects. GFAP provides glial-lineage context. Studies of body-fluid GFAP [17] and GFAP point-of-care testing [18] support biological interest in this marker, but do not directly validate tissue-flow GFAP measurements or the present semi-quantitative scoring categories. CD20, CD45, and CD19 identify hematopoietic or lymphoid populations; interpreting them as evidence of lymphomas requires population-level assessment and, where applicable, aberrancy or clonality evidence beyond an isolated positive marker [19,20,21]. Cytokeratin supports epithelial differentiation [22,23,24]. Thus, the components have a biological rationale, but the fluorescence thresholds and clinical context do not by themselves establish tumor-specific expression or validate the assigned weights.

All 36 glioma-spectrum records were assigned to the glioma interval, with 100% sensitivity and negative predictive value in this cohort. The interval is not a substitute for final integrated WHO CNS diagnosis. The score does not directly establish defining alterations such as IDH status and 1p/19q codeletion, or context-dependent molecular features such as TERT promoter mutation [2,25,26,27]. MGMT promoter methylation is principally a treatment-response biomarker and should not be grouped with WHO entity-defining alterations. The proposed classification may eventually complement intraoperative pathology, but such added value was not tested here.

The metastasis-oriented category had 10/11 sensitivity and 79/79 specificity. All 10 cytokeratin-positive records were metastases, so this part of the score was strongly marker-driven. Case 83 received zero cytokeratin points and remained below the metastasis interval. Sampling, assay performance, or non-epithelial differentiation are possible explanations in general [28,29].

The lymphoma-oriented category correctly classified 2/2 pathology-confirmed lymphomas; both were positive for CD45, CD19, and CD20 by flow cytometry. It also misclassified case 39, an atypical meningioma with borderline CD45 and CD20 signals. This illustrates a limitation of the current any-positive rule near the assay cutoff. Reactive lymphoid cells or nonspecific staining are possible explanations, but were not demonstrated in this case. Prior iFC studies support investigation of lymphoma-versus-glioblastoma discrimination [8], but positivity for an isolated lineage marker is insufficient evidence of lymphomas. The score supports broad classification and does not replace pathological diagnosis. The 95% exact interval for lymphoma sensitivity was 15.8–100.0%, so this arm remains exploratory and requires a larger series including reactive and inflammatory mimics.

The main classification overlap involved low-grade/meningioma-predominant records assigned to the glioma interval. The investigators confirmed the recorded diagnoses and measurements, and the component audit verifies how these scores crossed the 4/5 boundary. These remain classification errors of the evaluated rule. Biological heterogeneity and staining or processing effects are possible explanations, but were not specifically tested. Reference groups followed routine pathological diagnoses. Broad classification categories should therefore be distinguished from WHO grade. Future studies should evaluate whether revised grouping or additional lineage information improves discrimination without changing reference labels according to score performance.

The exploratory AUCs were 0.927 for low-grade/meningioma-predominant lesions, 0.935 for glioma, 0.994 for lymphoma, and 0.924 for metastasis. Low scores support the first category and high scores support metastasis, whereas glioma and lymphoma are interval-defined. All true glioma and lymphoma cases fell within their respective intervals, so their interval-evidence AUCs equal the mean of sensitivity and specificity at the fixed operating point. They therefore add no independent evidence of classification performance. The fixed-rule confusion matrix remains the primary result. Bootstrap intervals describe conditional sampling uncertainty and must not be interpreted as optimism correction or validation.

The component comparisons also constrain claims of added value. The full score showed only a small, uncertain accuracy difference from the marker-only rule, and removal of DNA or low-DNA-content points did not change any classifications in this cohort. These findings do not show that the measurements lack biological or clinical value; they show that their incremental contribution was not demonstrated with these cases, recorded points, and fixed intervals. A prospective comparison with prespecified marker-only and pathology-based approaches is required.

The present work aligns with, but differs from, the Japanese iFC literature. Shioyama, Muragaki, and colleagues developed malignancy index as a rapid DNA-content-based parameter for identifying glioma tissue and estimating histopathological grade [5]. Subsequent studies extended iFC to prognostic value in low-grade gliomas and glioblastoma, meningioma malignancy assessment, and lymphoma-versus-glioblastoma differentiation [6,7,8,9]. Most recently, Koriyama and colleagues integrated iFC-derived features with preoperative imaging and machine learning to predict diffuse glioma molecular subtypes in real time in 288 patients, achieving 76.0% overall accuracy, a macro-average ROC-AUC of 0.88, and a micro-average ROC-AUC of 0.89 [30]. The Kefallinia Score follows a different strategy: rather than predicting molecular subtype, it offers a transparent rule-based taxonomy for broad intraoperative diagnostic orientation. These approaches should be viewed as complementary.

There are several limitations of the current study. The score was defined before specimen collection, but its performance was assessed retrospectively in a cohort restricted to 90 complete-panel cases. Such inclusion may limit applicability to incomplete or failed assays. The reference categories are broad pathological groups rather than detailed integrated WHO entities. The low-DNA-content component was not independently validated as necrosis, and lymphoma was represented by only two cases. Marker-associated weights dominate some assignments, and incremental accuracy over a simpler marker rule was not established. Finally, complete-panel turnaround time, prospective feasibility, interoperator or intercenter reproducibility, comparison with routine intraoperative pathology, and effects on surgical decisions or outcomes were not evaluated.

Prospective validation is the next necessary step. Ideally, the Kefallinia Score should be tested in real time during neurosurgical procedures, with prespecified scoring rules, standardized gating, and blinded comparison against frozen section and final integrated diagnosis. Endpoints should include concordance with final diagnosis, time to result, impact on intraoperative decision-making, agreement with frozen section, margin-assessment value, and reproducibility across operators and centers. Future work could compare cell-cycle/DNA analysis alone, marker-assisted Kefallinia Score, and machine-learning-enhanced models.

## 5. Conclusions

In conclusion, the predefined Kefallinia Score provides an interpretable framework for organizing DNA-content and marker-derived information into broad intracranial tumor categories. It correctly classified 81/90 cases in the retrospective complete-panel cohort. These findings support its initial classification performance and further evaluation as an adjunct to intraoperative pathological assessment. Independent prospective studies are required to establish reproducibility, complete-panel feasibility and added clinical value.

## Figures and Tables

**Table 1:** Kefallinia Score components and category assignment.

Component/Score Range	Scoring Rule/Assigned Category
DNA-index component	DNA index < 0.9 or >1.1: 4 points; 0.9 ≤ DNA index ≤ 1.1: 0 points
Tumor-index component	<6%: 0 points; 6–10%: 2 points; >10%: 3 points
Low-DNA-content component	Operational low-DNA-content fraction >10%: 2 points; ≤10%: 0 points.
CD20/CD45/CD19 component	At least one of CD20/CD45/CD19 with r ≥ 2: 15 points; otherwise: 0 points. r is marker/control geometric mean fluorescence.
Cytokeratin component	Cytokeratin r ≥ 2: 30 points; r < 2: 0 points r is marker/control geometric mean fluorescence.
GFAP component	GFAP relative fluorescence: r < 2, absent, 0 points; 2 ≤ r ≤ 4, low, 1 point; 4 < r ≤ 10, medium, 2 points; r > 10, high, 5 points. r is marker/control geometric mean fluorescence.
0–4	Low-grade/meningioma-predominant lesion
5–14	Glioma spectrum
15–29	Lymphoma-oriented profile
≥30	Metastasis-oriented profile

**Table 2:** One-vs-rest classification performance. Entries give numerator/denominator, percentage, and exact binomial 95% confidence interval. PPV, positive predictive value; NPV, negative predictive value. Predictive values reflect the diagnostic distribution of this selected cohort.

Category	Sensitivity	Specificity	PPV	NPV
Low-grade/mng	33/41; 80.5% (65.1–91.2)	48/49; 98.0% (89.1–99.9)	33/34; 97.1% (84.7–99.9)	48/56; 85.7% (73.8–93.6)
Glioma	36/36; 100.0% (90.3–100.0)	47/54; 87.0% (75.1–94.6)	36/43; 83.7% (69.3–93.2)	47/47; 100.0% (92.5–100.0)
Lymphoma	2/2; 100.0% (15.8–100.0)	87/88; 98.9% (93.8–100.0)	2/3; 66.7% (9.4–99.2)	87/87; 100.0% (95.8–100.0)
Metastasis	10/11; 90.9% (58.7–99.8)	79/79; 100.0% (95.4–100.0)	10/10; 100.0% (69.2–100.0)	79/80; 98.8% (93.2–100.0)

## Data Availability

De-identified component points, reference groups, score formulas, classification audits and statistical summaries are provided in Supplementary Tables S1–S6. The laboratory workflow and representative marker histograms are provided in Supplementary Methods S1 and Figure S1.

## Supplementary Materials

The following supporting information can be downloaded at: https://www.mdpi.com/article/10.3390/cancers18213547/s1, Supplementary Table S1. De-identified component points and classification audit. Supplementary Table S2. Score distributions by reference group. Supplementary Table S3. Fixed four-class confusion matrix. Supplementary Table S4. Misclassified cases. Supplementary Table S5. Exploratory one-vs-rest ROC analysis. Supplementary Table S6. Exploratory fixed-rule component comparisons. Supplementary Figure S1. Representative marker histograms in glioma, metastasis and lymphoma specimens. Supplementary Methods S1. The Kefallinia Score laboratory workflow.
