# Supplementary material for: The Kefallinia Score: Development and Apparent Performance Assessment of a Flow Cytometry-Based Intraoperative Classification System for Intracranial Tumors
Source: Cancers (Basel). 2026 Nov 4;18(21):3547. doi: 10.3390/cancers18213547 (PMC13761862; doi:10.3390/cancers18213547)
Supplement: Supplementary file 1 [file cancers-18-03547-s001.zip › Supplementary_Methods_and_Figure_S1.pdf]

Supplementary Methods

The Kefallinia Score

This supplement summarizes the published laboratory workflow underpinning the present analysis and the study-specific scoring definitions. The components, weights and category intervals were defined before specimen collection; the finalized 90-case table was used for retrospective performance assessment.

Supplementary Methods S1

Tissue preparation and DNA analysis

The Ioannina workflow used small fresh tumor specimens, mechanical dissociation with a Medimachine System and CellTrics filtration to produce a single-cell suspension [1]. Tissue was minced in phosphate-buffered saline (PBS) for 1 minute, adjusted to approximately 10^6 cells/mL and stained with propidium iodide at 125 µg/mL for 3 minutes before acquisition [3]. Normal PBMCs isolated by Ficoll separation and stained under the same conditions provided the diploid reference. Analysis used a FACSCalibur cytometer and CellQuest V3 software [3]. At least 5,000 cells were analyzed [2].

Cell-cycle indices and low-DNA-content component

Manual DNA-histogram gates defined G0/G1, S and G2/M fractions [1]. TI was the percentage in S plus G2/M. DI was the ratio of the tumor G0/G1 geometric mean fluorescence to the normal diploid reference. The score assigned 4 points for DI<0.9 or DI>1.1 and 0 otherwise. TI received 0 points for <6%, 2 for 6–10% and 3 for >10%. Retained events below the G0/G1 DNA population, after exclusion of events interpreted as debris, formed the operational low-DNA-content component. Its recorded fraction received 2 points if >10% and 0 otherwise. This empirically defined component may indicate necrosis. The marker was added to include a quantitative analysis of low DNA content events.

Marker analysis and fluorescence categories

Our analysis was based on previous published method compared FITC-conjugated anti-human cytokeratin (clone CAM5.2, BD Biosciences) with mouse IgG1-FITC isotype control and inspected the individual and overlaid fluorescence distributions [3]. For the present score, relative expression was r = marker-stained geometric mean fluorescence / isotype-control geometric mean fluorescence. CD45-FITC (clone 2D1, BD Biosciences), CD19-FITC (clone SJ25C1, BD Biosciences), CD20-FITC (clone L27, BD Biosciences) or cytokeratin positivity was defined at r≥2. Positivity for any of the three lymphoid markers received 15 points; cytokeratin positivity received 30. GFAP-Alexa Fluor 488 (clone 2E1.E9, Biolegend) received 0 points for r<2, 1 for 2≤r≤4, 2 for 4<r≤10 and 5 for r>10. Representative histograms are shown in Supplementary Figure S1.

Interpretation and scope

Laboratory assessment preceded disclosure of final pathology. Reference groups followed the final routine pathological diagnoses. Total-score intervals were 0–4, 5–14, 15–29 and ≥30 for low-grade/meningioma-predominant, glioma-spectrum, lymphoma-oriented and metastasis-oriented profiles, respectively. The verified component points were retained. Marker signals support classification and do not independently establish a neoplastic lineage. Published DNA-analysis timing is not a measured turnaround time for the full panel.

Supplementary Figure S1

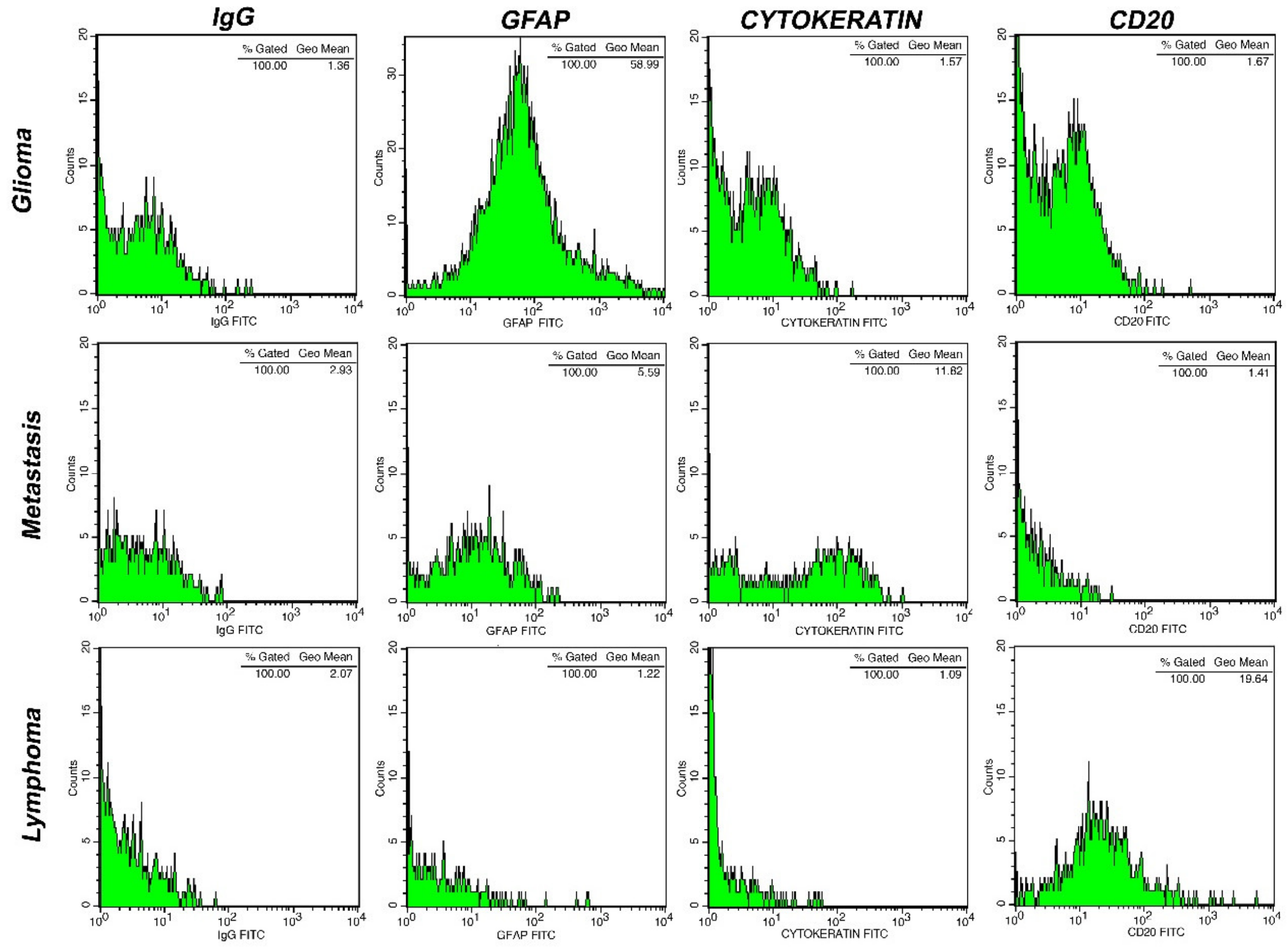

Representative marker histograms in glioma, metastasis and lymphoma specimens. Rows show the three illustrated specimens; columns show isotype IgG, GFAP, cytokeratin and CD20. The horizontal axis is FITC fluorescence intensity on a logarithmic scale and the vertical axis shows event counts. Each inset displays the geometric mean fluorescence and the percentage within the displayed gate; "100% gated" does not imply 100% marker-positive tumor cells. Relative expression is calculated separately within each row against its IgG control. Glioma GFAP is 59.99/1.36=44.1 (high); metastasis cytokeratin is 11.82/2.93=4.0 (positive); lymphoma CD20 is 19.64/2.07=9.5 (positive). The illustrated metastasis GFAP ratio is 5.59/2.93=1.91 (absent). These examples illustrate assay interpretation.

References

1. Vartholomatos G, et al. Assessment of Gliomas' Grade of Malignancy and Extent of Resection Using Intraoperative Flow Cytometry. Cancers. 2023;15:2509. https://doi.org/10.3390/cancers15092509

2. Alexiou GA, et al. Intraoperative Flow Cytometry for the Evaluation of Meningioma Grade. Current Oncology. 2023;30:832–838. https://doi.org/10.3390/curroncol30010063

3. Markopoulos GS, et al. Intraoperative Flow Cytometry Upon and Beyond the Cell Cycle: A Case Study of the Characterization of a Bone Metastasis. Journal of Molecular Pathology. 2023;4:225–233. https://doi.org/10.3390/jmp4040020
